# HBeAg mediates inflammatory functions of macrophages by TLR2 contributing to hepatic fibrosis

**DOI:** 10.1186/s12916-021-02085-3

**Published:** 2021-10-15

**Authors:** Xiaoyu Xie, Huanran Lv, Chenxi Liu, Xiaonan Su, Zhen Yu, Shouyang Song, Hongjun Bian, Miaomiao Tian, Chengyong Qin, Jianni Qi, Qiang Zhu

**Affiliations:** 1 Shandong Provincial Hospital Affiliated to Shandong First Medical University, Jinan, Shandong 250021 People’s Republic of China; 2 Shandong Provincial Hospital, Cheeloo College of Medicine, Shandong University, Jinan, Shandong 250021 People’s Republic of China; 3 Shandong Provincial Engineering and Technological Research Center for Liver Diseases Prevention and Control, Jinan, Shandong 250021 People’s Republic of China; 4 The First Affiliated Hospital of Xinjiang Medical University, Urumqi, Xinjiang, 830054 People’s Republic of China

**Keywords:** HBeAg, Macrophage, Hepatic stellate cell, Inflammation, Fibrosis

## Abstract

**Background:**

We and others have confirmed activation of macrophages plays a critical role in liver injury and fibrogenesis during HBV infection. And we have also proved HBeAg can obviously induce the production of macrophage inflammatory cytokines compared with HBsAg and HBcAg. However, the receptor and functional domain of HBeAg in macrophage activation and its effects and mechanisms on hepatic fibrosis remain elusive.

**Methods:**

The potentially direct binding receptors of HBeAg were screened and verified by Co-IP assay. Meanwhile, the function domain and accessible peptides of HBeAg for macrophage activation were analyzed by prediction of surface accessible peptide, construction, and synthesis of truncated fragments. Furthermore, effects and mechanisms of the activation of hepatic stellate cells induced by HBeAg-treated macrophages were investigated by Transwell, CCK-8, Gel contraction assay, Phospho Explorer antibody microarray, and Luminex assay. Finally, the effect of HBeAg in hepatic inflammation and fibrosis was evaluated in both human and murine tissues by immunohistochemistry, immunofluorescence, ELISA, and detection of liver enzymes.

**Results:**

Herein, we verified TLR-2 was the direct binding receptor of HBeAg. Meanwhile, C-terminal peptide (122-143 aa.) of core domain in HBeAg was critical for macrophage activation. But arginine-rich domain of HBcAg hided this function, although HBcAg and HBeAg shared the same core domain. Furthermore, HBeAg promoted the proliferation, motility, and contraction of hepatic stellate cells (HSCs) in a macrophage-dependent manner, but not alone. PI3K-AKT-mTOR and p38 MAPK signaling pathway were responsible for motility phenotype of HSCs, while the Smad-dependent TGF-β signaling pathway for proliferation and contraction of them. Additionally, multiple chemokines and cytokines, such as CCL2, CCL5, CXCL10, and TNF-α, might be key mediators of HSC activation. Consistently, HBeAg induced transient inflammation response and promoted early fibrogenesis via TLR-2 in mice. Finally, clinical investigations suggested that the level of HBeAg is associated with inflammation and fibrosis degrees in patients infected with HBV.

**Conclusions:**

HBeAg activated macrophages via the TLR-2/NF-κB signal pathway and further exacerbated hepatic fibrosis by facilitating motility, proliferation, and contraction of HSCs with the help of macrophages.

**Supplementary Information:**

The online version contains supplementary material available at 10.1186/s12916-021-02085-3.

## Background

Hepatitis B is a global public health problem, since patients infected with hepatitis B viruses (HBV) are at increased risk of progression to chronic active hepatitis, cirrhosis, and hepatocellular carcinoma (HCC) [1, 2]. After HBV entry into hepatocytes and begin to replicate, virus-related proteins can be detected in the liver and peripheral blood. HBV-related proteins are mainly composed of hepatitis B surface antigen (HBsAg), hepatitis B core-related antigen (HBcrAg), and HBx proteins [3]. HBcrAg consists of three proteins coded by the precore/core region including hepatitis B core antigen (HBcAg), hepatitis B e antigen (HBeAg), and a 22-kDa precore protein. In clinical practice, HBsAg has been estimated as a surrogate marker of HBV infection or intrahepatic viral replicative activity. Several studies reveal that HBsAg plays a vital role in promoting persistent HBV infection, whereas others indicate HBsAg and HBcAg can also induce immune response and facilitate liver injury [3–5]. HBx proteins can sensitize hepatocytes to carcinogenic factors and lead to HCC by deregulating cell apoptosis and proliferation control [6]. Moreover, the level of serum HBeAg is associated with viral replication, infectivity, inflammation, severity of disease, and response to antiviral therapy [7]. However, the function of virus-related proteins to regulate the immune response and the underlying mechanisms have not been completely elucidated.

The persistence, clearance, and pathogenesis of HBV are closely related to the interaction between the innate immune system and various viral proteins. Macrophages, as an important component of the innate immune system, play a crucial role in detecting HBV and regulating inflammation-induced liver injury. Inflammatory cytokines from macrophages inhibit virus replication, but also result in damage of hepatocytes simultaneously [8]. On the other hand, viral proteins abrogate immune responses of macrophages leading to immune tolerance or establishment of chronic HBV infection [9]. Our recent work has proved that, compared with HBcAg and HBsAg, HBeAg is the most important element in HBV-associated antigens inducing macrophage activation, and HBeAg-induced miR-155 accelerate liver injury by promoting inflammatory cytokine production [10]. Yet, the receptors by which macrophages recognize HBeAg and the molecular mechanism of their activation are still a mystery.

Toll-like receptor (TLR) family is one of the best-known pattern recognition receptor (PRR) families and responsible for recognizing diverse molecules derived from pathogens and damaged host cells [11]. For example, lipoproteins and lipopolysaccharides, the major components of the outer membrane of bacteria, are the best-known ligands for TLR-2 and TLR-4. Moreover, intracellular life-encoding molecules, such as DNA and RNA, are recognized by TLR-9 and TLR-3/7/8, respectively. Recently, TLR ligands are still increasing in number and cover a variety of microbial components. Chang et al. find TLR-2 may also play a key role in recognizing hepatitis C virus core and NS3 proteins, thereby activating macrophages [12]. Besides, Enterovirus-71 virus-like particles induce the activation and maturation of human monocyte-derived dendritic cells by activating TLR-4 [13]. Whether HBeAg could also be recognized by macrophages through TLR-dependent signal pathway remains to be further explored.

In chronic HBV infection, progressive hepatocellular damage induced by persistent immune response, accompany with extensive tissue remodeling and vascular disorganization, will ultimately culminate into fibrosis and cirrhosis [14]. It is well-known that activated hepatic stellate cells (HSCs) remain central effector cells driving its progression, which undergo a phenotypic transdifferentiation into highly proliferative, contractile, chemotactic, and fibrogenic myofibroblasts [15]. In hepatic microenvironment, extracellular signals from the surrounding cells can act in a paracrine manner to promote the activation of HSCs. Macrophages are one of the major regulators of fibrosis progression and remodeling of deposited extracellular matrix (ECM). Accumulating evidence indicates that activated macrophages can produce pro-fibrotic mediators, such as growth factors, cytokines, and chemokines, etc, contributing to the activation and recruitment of HSCs, and further accelerate the deposition of ECM. On the other hand, activated HSCs also secrete chemokines to attract macrophages, leading to the amplification of this pathological process [16]. Our previous results have established that macrophages activated by HBeAg increase the expression and secretion of multiple cytokines [10]. However, it is still unclear whether HBeAg can activate HSCs to promote the occurrence and progression of hepatic fibrosis directly or in a macrophage-dependent manner.

In this study, we uncovered that HBeAg affected the expression of TLRs in macrophages, thereby regulating the innate immune response. In accordance with the high expression of TLR-2, HBeAg acted as a new ligand of TLR-2 to induce growth factors, cytokines, and chemokines via NF-κB-mediated signaling pathway, and its C-terminal peptide (122-143 aa.) is critical for the activation of macrophages. In addition, we found that HBeAg promoted the proliferation, contraction, and motility of HSCs in a macrophage-dependent manner, demonstrating a novel mechanism for the progression of hepatic fibrosis and its related complications. Consistent with the above-mentioned results, clinical data indicated the level of HBeAg is associated with grades of inflammation, macrophage infiltration, and the stage of fibrosis in patients infected with HBV.

## Methods

### Patients

Two hundred twelve patients, including 61 subjects with acute hepatitis B (AHB) and 151 subjects with chronic hepatitis B (CHB), were recruited from Shandong Provincial Hospital Affiliated to Shandong First Medical University from May 2017 to March 2018. AHB and CHB were diagnosed based on EASL 2017 clinical practice guidelines on the management of hepatitis B virus infection [17]. The inclusion criteria for this study were (1) ≥18 years old and (2) the presence of hepatitis B surface antigen. The exclusion criteria were (1) co-infection with HCV, HIV, or other chronic liver diseases; (2) a mean daily consumption of > 40 g of alcohol for men and > 20 g of alcohol for women; (3) previous liver surgery or liver transplantation; and (4) antiviral therapy or immunosuppressive drugs within 3 months. Verbal or written informed consents were obtained from each of the patients/guardians before being included in this study, and this study was approved by the Ethics Committee of Shandong Provincial Hospital Affiliated to Shandong First Medical University. Basic information of patients is summarized in Table 1. Additionally, complications of hepatic fibrosis and cirrhosis were detected by proficient endoscopists and ultrasound experts respectively [18].

**Table 1:** Clinical characteristics of enrolled subjects

Variable	CHB (*n* = 151)	AHB (*n* = 61)	*P*
Age (years)	54.41±9.69	36.54±11.79	**<0.001**
Sex (male)	121 (80.10%)	45 (73.80%)	0.309
ALT (IU/L)	36.00 (25.00, 49.00)	1325.00 (774.70, 2345.50)	**<0.001**
AST (IU/L)	32.00 (20.00, 50.00)	897.00 (375.00, 1351.50)	**<0.001**
HBeAg (COI)	0.14 (0.11, 1.20)	20.03 (5.85, 148.88)	**<0.001**
HBsAg (COI)	5611.50 (879.15, 6902.00)	3666.61 (1381.50, 5918.0)	0.426
HBV DNA (log_10_ IU/mL)	3.27±1.83	4.61±1.34	**<0.001**
Stage of fibrosis		NS	NS
0	13 (8.61%)		
1	14 (9.27%)		
2	44 (29.14%)		
3	20 (13.25%)		
4	60 (39.73%)		
Grade of inflammation		NS	NS
0	10 (6.62%)		
1	92 (60.93%)		
2	29 (19.21%)		
3	20 (13.25%)		

Data are reported as mean ± SD, median (interquartile range), or *n* (%)

Abbreviations: *IU* international units; *COI* cut-off index; *LS* liver stiffness; *ALT* alanine aminotransferase; *AST* aspartate aminotransferase; *HBeAg* hepatitis B e antigen; *HBsAg* hepatitis B surface antigen; *HBV DNA* hepatitis B DNA quantification

### Animal experiments

Male Balb/c mice were purchased from Beijing Vital River Laboratory Animal Technology Co., Ltd. (Beijing, China) and housed in a specific pathogen-free environment. All experiments were conducted with mice between 6 and 8 weeks of age in compliance with the Scientific Investigation Board of the Shandong Provincial Hospital Affiliated to Shandong First Medical University. To examine the role of HBeAg in vivo, mice (5 mice in each group) were injected with recombinant HBeAg 40 μg or received the equivalent volume of PBS via tail vein, then were sacrificed at 4, 8, 12, and 24 h respectively. To study the effect of HBeAg in the progress of hepatic fibrosis, mice were treated with a single intraperitoneal injection of olive oil or CCl_4_ (1 ml/kg in olive oil) at day 1. At day 2 and day 3, CCl_4_-treated mice received intravenous administration of HBeAg 40 μg or the same volume of PBS (*n* = 5 per group). At day 4, all mice were sacrificed, and the liver and blood were harvested and frozen for further analyses. Monocyte depletion was achieved by way of intraperitoneal injection of 200 μl clodronate-liposome or control-liposome before CCl_4_ or HBeAg treatment. To validate the effect of TLR-2 in vivo, C29 was dissolved and diluted to appropriate doses, then injected intraperitoneally (1.3 μmol/g) 1 h before HBeAg treatment, with the same volume of dissolving reagent (10% DMSO/40% PEG300/5% Tween-80/45% saline) as the vehicle group.

### Cell culture, reagents, and antibodies

Mouse macrophage cell lines RAW264.7 (ATCC, Rockville, MD, USA) and human stellate cell lines LX-2 (Procell, Wuhan, China) were cultured in DMEM (Gibco- BRL, Grand Island, NY, USA) containing 10% (vol/vol) FBS (Gibco® Sera, AUS). Human monocyte cell lines THP-1, U937 (ATCC, Rockville, MD, USA) were cultured in RPMI 1640 medium (Gibco; Thermo Fisher Scientific, Inc.) supplemented with 10% FBS (Gibco® Sera, AUS). Mouse primary hepatic stellate cells and Kupffer cells were obtained and cultured based on the previous description [4, 19]. Human monocyte-derived macrophages (hMDM) were differentiated from peripheral blood mononuclear cells (PBMCs) of healthy human blood donors using Ficoll-Paque density gradient centrifugation [12]. All cells were incubated at 37 °C in an incubator with 5% CO_2_.

HBeAg (ab91273) and HBcAg (ab119441) were purchased from Abcam (Abcam, Cambridge, UK). C29 (TLR-2 inhibitor, HY-100461), resatorvid (TLR-4 inhibitor, HY-11109), hydroxychloroquine sulfate (TLR-7/9 inhibitor, HY-B1370), CU-CPT9b (TLR-8 inhibitor, HY-112051), SB202190 (p38 MAPK inhibitor, HY-10295), SP600125 (JNK inhibitor, HY-12041), PD98059 (ERK1/2 inhibitor, HY-12028), tofacitinib (JAK1/2/3 inhibitor, HY-40354), (E)-SIS3 (Smad3 inhibitor, HY-13013), and dactolisib (PI3K/mTOR inhibitor, HY-50673) were acquired from MCE (MedChemExpress, Pudong New Area, Shanghai, China). PMA (phorbol 12-myristate 13-acetate, ab120297) was purchased from Abcam (Abcam, Cambridge, UK). Blocking antibody for TLR-3 (ab17264), TLR-2 (ab16894), and TLR-4 (ab30667) were purchased from Abcam (Abcam, Cambridge, UK). Primary antibodies for GAPDH (60004-1-lg), β-actin (66009-1-Ig), and TLR-6 (22240-1-AP) were purchased from Proteintech Group, Inc. (Proteintech, Wuhan, China). Primary antibodies for TLR-1 (abs135699) were purchased from Absin Bioscience Inc. (Absin, Shanghai, China). Primary antibodies for P65(#8242), p-P65 (#3033), GST (#3368, #2624 and #2625), α-SMA (#19245), Collagen1A1 (#91144), p-FOXO3a (#9466), p-Smad3 (#9520), p-Smad2(#18338), mTOR (#2983), p-JNK (#4668), p-P38 (#4511), P38 (#8690), p-ERK (#4370), ERK (#4695), p-PI3K (#4228), and PI3K (#4257) were obtained from cell signaling (Cell-Signaling Technology, Boston, USA). Primary antibodies for TLR-2 (ab209217), F4/80 (ab6640), Fibronectin (ab268021), CD68 (ab213363), FOXO3a (ab109629), Smad3 (ab40854), Smad2 (ab33875), p-STAT5 (ab32364), STAT5 (ab32043), p-STAT3 (ab76315), STAT3(ab68153), p-mTOR (ab109268), JNK (ab179461), p-AKT1 (ab108266), and AKT1 (ab108202) were purchased from Abcam (Abcam, Cambridge, UK). Corresponding HRP-conjugated secondary antibodies were purchased from Santa Cruz (Santa Cruz Biotechnology, Dallas, TX, USA). Alexa Fluor® 488 and 594-conjugated secondary antibodies for immunofluorescence were purchased from Abcam (Abcam, Cambridge, UK). Recombinant HBeAg domains (ArD, ΔRP-E, Δ1-17-E, Δ29-96-E, and Δ122-143-E) were obtained from Dia-An Biotech (Dia-An Biotech, Wuhan, China). Clophosome-A clodronate liposomes (Anionic, 7 mg/ml, F70101C-A) and control liposomes (F70101-N) were purchased from FormuMax (Sunnyvale, CA, USA).

### RNA interference

Small interfering (si) RNA targeting the human TLR-2 (seq-1: 5′-TTTGATGACTGTACCCTTAAT-3′; seq-2: 5′-GGAAGATAATGAACACCAA-3′; seq-3: 5′-GGCTTCTCTGTCTTGTGAC-3′), mouse TLR-1 (seq-1: 5′-CCGTCCCAAGTTAGCCCATTT-3′; seq-2: 5′-GCCTTCAGGATGTTCAATTAT-3′; seq-3: 5′-CATCCTCTCATTGTCCAAGCT-3′), mouse TLR-6 (seq-1: 5′-CAATACCACCGTTCTCCATTT-3′; seq-2: 5′-GGAATGGTTTGAAGAACTT-3′; seq-3: 5′-CCGGTGGAGTACCTCAATATT-3′), mouse TLR-3 (seq-1: 5′-GCTGAGCAGTTTGAATATA-3′; seq-2: 5′-CCACCTACCAACTTTACAA-3′; seq-3: 5′-CACTCCACATCATTATTAT-3′), human TLR-1 (seq-1:5′-GCTCATTTGAATATCAGCAA-3′; seq-2: 5′-ACTTAAGGGCAGCCATTAATA-3′; seq-3: 5′-GATGAAGTCTCTGCAACAATT-3′), human TLR-6 (seq-1:5′-GGTCTTATTCATGTTCCAA-3′; seq-2: 5′-CAAAGAACCTATTGTTAAA-3′; seq-3: 5′-GGTGCTTACAACTGACTAA-3′) sequences, and negative control siRNA were purchased from Genepharma (Pudong new area, Shanghai, China). RNAis were added to cells accompany with the lipofectamine MAX (Invitrogen, Camarillo, CA, USA) based on the manufacturer’s protocol. After interfering for 48 h, protein knockdown efficiency was analyzed by western blot.

### Quantitative real-time PCR (qRT-PCR), library preparation, and sequencing of RNA

Total RNA extraction, qRT-PCR, and RNAseq were performed as we previously described [10]. Sequences of the primers for qRT-PCR are listed in Table 2. The expression profile of growth factors, cytokines, chemokines, and TLRs were selected, and the corresponding heat map was drawn.

**Table 2:** Primers used for quantitative real-time PCR

Gene	Forward primer	Reverse primer
Homo-IL-6	CCTGAACCTTCCAAAGATGGC	TTCACCAGGCAAGTCTCCTCA
Homo-TNF-α	GAGGCCAAGCCCTGGTATG	CGGGCCGATTGATCTCAGC
Homo-COL1A1	TAAAGGGTCACCGTGGCTTC	GGGAGACCGTTGAGTCCATC
Homo-α-SMA	ATGCCTCTGGACGCACAACT	CCCGGACAATCTCACGCTCA
Homo-Fibronectin	GGGTCTCCTCCCAGAGAAGT	GTTGGGGAAGCTCGTCTGTC
Homo-GAPDH	GCACCGTCAAGGCTGAGAAC	TGGTGAAGACGCCAGTGGA
Mus-IL-6	GCCTTCTTGGGACTGATGCT	GCCATTGCACAACTCTTTTCTCA
Mus-TNF-α	CGGGCAGGTCTACTTTGGAG	ACCCTGAGCCATAATCCCCT
Mus-IFN-γ	GGGTTGTATCTGGGGGTGGG	GTCACTGCAGCTCTGAATGTTTCTT
Mus-IL-10	GCTCTTGCACTACCAAAGCC	CTGCTGATCCTCATGCCAGT
Mus-TGF-β1	AGGGCTACCATGCCAACTTC	CCACGTAGTAGACGATGGGC
Mus-Ccl-2	CACTCACCTGCTGCTACTCA	GCTTGGTGACAAAAACTACAGC
Mus-Fibronectin	ACCTTGATCTCCCAAGCACG	CGTCAGGTGCTGTAGTCTGT
Mus-Col1A1	CCCTGGTCCCTCTGGAAATG	GGACCTTTGCCCCCTTCTTT
Mus-α-SMA	TTCGTGACTACTGCCGAGC	GTCAGGCAGTTCGTAGCTCT
Mus-GAPDH	TGTCTCCTGCGACTTCAACA	GGTGGTCCAGGGTTTCTTACT
Homo-TLR-1	GGAGGCAATGCTGCTGTTCA	GCCCAATATGCCTTTGTTATCCTG
Homo-TLR-2	CTCCCAGCAGGAACATCTGCTA	CCAGGAATGAAGTCCCGCTTA
Homo-TLR-3	CCTGATGAAATGTCTGGATTTGGA	AACAGTGCACTTGGTGGTGGAG
Homo-TLR-4	CTGGGTGTGTTTCCATGTCTCA	TGCGGACACACACACTTTCAAATA
Homo-TLR-5	GATGCTACTGACAACGTGGCTTC	AAGCTGGGCAACTATAAGGTCAGG
Homo-TLR-6	CTGTCTGCATTAGCCCTTCCTTG	TGTGGAAGAATGTGCCGTTTG
Homo-TLR-7	TTCAACCAGACCTCTACATTCCATT	GCAGTCCACGATCACATGGTT
Homo-TLR-8	CTTTGCAGAGGCTAATGGATGAGAA	CTGCCGTAGCCTCAAATACTGAGAA
Homo-TLR-9	CCGTGACAATTACCTGGCCTTC	CAGGGCCTTCAGCTGGTTTC
Mus-TLR-1	TGACCTGCCCTGGTATGTGAG	GGCAGAATCATGCCCACTGTA
Mus-TLR-2	GAGCATCCGAATTGCATCACC	CCCAGAAGCATCACATGACAGAG
Mus-TLR-3	AAATCCTTGCGTTGCGAAGTG	TCAGTTGGGCGTTGTTCAAGA
Mus-TLR-4	CATGGATCAGAAACTCAGCAAAGTC	CATGCCATGCCTTGTCTTCA
Mus-TLR-5	GCTTGGAACATATGCCAGACACA	AAAGGCTATCCTGCCGTCTGAA
Mus-TLR-6	AATGGTACCGTCAGTGCTGGAAATA	TGGCTCATGTTGCAGAGGCTA
Mus-TLR-7	CTTTGCAACTGTGATGCTGTGTG	ACCTTTGTGTGCTCCTGGACCTA
Mus-TLR-8	ACGGCTTGCCATCTTGGTC	AGTGGCAAATGTTCTTAGGGATTGA
Mus-TLR-9	GAGACCCTGGTGTGGAACATC	ACTGCAGCCTGTACCAGGAG

### Co-immunoprecipitation (Co-IP) and western blot analysis

According to the manufacturer’s protocols, Co-IP was performed using a Capturem IP & Co-IP Kit (Takara, Kusatsu, Japan). Briefly, per 10^6^ cells were lysed by 200 μl lysis/equilibration buffer mixed with protease inhibitor cocktail on ice for 30 min. Cell lysis supernatant was obtained after centrifuging at 17,000g for 10 min. Then, the supernatant or mixed recombinant proteins were incubated with GST or TLR-2 antibody or isotype IgG (Cell Signalling Technology, Boston, MA, USA) overnight at 4°C. In the second day, samples (pre-incubated with antibody) were centrifuged using an equilibrated spin column and washed with 100 μl Wash Buffer. Eluted proteins were analyzed by western blot using GST or TLR-2 primary antibody. Recombinant human TLR-2 (H-TLR-2, 2616-TR) and mouse TLR-2 (M-TLR-2, 1530-TR) were purchased from R&D Systems (Minneapolis, MN, USA). Western blot assay was performed as we described previously [10].

### Enzyme-linked immunosorbent assay (ELISA) and liver enzyme assays

Based on the manufacturer’s instructions, mouse IL-6 (KMC0061, Invitrogen, Carlsbad, CA, USA), mouse TNF-α (KMC3011, Invitrogen, Carlsbad, CA, USA), mouse IFN-β (439407, BioLegend, Inc. San Diego, CA), human IL-6 (Proteintech, Wuhan, China), and human TNF-α (Proteintech, Wuhan, China) in cell-culture supernatants were measured by commercially available ELISA kits. Serum alanine aminotransferase (ALT) and aspartate aminotransferase (AST) concentrations were determined using commercially available kits purchased from Nan Jing Jan Cheng Biochemical Institute (Nanjing, China).

### Histopathologic evaluation and immunohistochemistry

Liver specimens from biopsies or surgeries were fixed with formalin then embedded in 5-μm-thick paraffin sections. Blinded to the clinical data, a histopathologic assessment was evaluated by a senior hepatologist for all CHB patients. The METAVIR score system was applied for differentiating hepatic fibrosis and inflammatory activity via Masson’s trichrome and hematoxylin-eosin staining [20, 21]. Besides, immunohistochemical staining was detected as previously described [22]. The positive area rates were assessed by Image-Pro Plus 5.0.

### Immunofluorescence

NF-κB nuclear translocation and HBeAg cellular localization were detected with monoclonal antibody to P65, and GST tag. For NF-κB nuclear translocation, Alexa Fluor® 488 or 594-conjugated secondary antibodies (Abcam, Cambridge, UK) were incubated for 2 h. Nuclei were stained with 4′,6-diamidino-2-phenylindole (DAPI, Abcam, Cambridge, UK). Double immunofluorescence staining of α-SMA and F4/80 was conducted on liver cryosections and incubated overnight with primary rat anti-mouse F4/80 (Abcam, Cambridge, UK) and rabbit anti-mouse-α-SMA (Cell-Signaling Technology, Boston, USA) and then with secondary antibodies and DAPI. Immunolocalization of Desmin and ki67 was performed on liver cryosections and incubated with primary rat anti-mouse ki67 (Thermo Fisher Scientific, Waltham, MA) and rabbit anti-mouse-Desmin (Cell-Signaling Technology, Boston, USA). Images were captured using a fluorescence microscope (Olympus BX63, Tokyo, Japan) or confocal fluorescence microscopy (Leica TCS SP8).

### Preparation of the conditioned medium

The medium collected from control and activated macrophages was defined as the conditioned medium (CM). After U937 cells were cultured in the presence of PMA (Abcam, Cambridge, UK) at a final concentration of 50 ng/ml for 24 h, most of the suspended cells differentiated into macrophages and attached to the plate bottom. Next, the adherent cells were washed twice with PBS and cultured with a serum-free medium. Macrophages were maintained in a serum-free medium throughout the experimental period as control or incubated with HBeAg for 24 h to activate macrophages. CM collected from control (CM-C) and activated macrophages (CM-E) was filtered with a 0.45-mm membrane filter before being added to LX-2 cells.

### Hepatic stellate cell migration assay

HSC migration was measured by transwell assays. About 10^4^ HSCs were suspended in a medium supplemented with 1% FBS on the upper chambers of the 24-well transwell plate (pore diameter 8 μm). Lower chambers were supplemented with 10% FBS and (a) serum-free medium which has been incubated at 37 °C in an incubator with 5% CO_2_ for 24 h to keep the same conditions as CM, (b) serum-free medium with HBeAg (2 μg/ml), (c) CM-C, and (d) CM-E. Transwell plates were maintained in an incubator for 24 h and cell migration was estimated by mean values of five randomly chosen fields captured on the lower surface of the filters.

### Cell proliferation assay

The relative proliferation rates of the hepatic stellate cell were determined by Cell Counting Kit-8 (CCK-8) assay (Vazyme, Nanjing, China). HSCs were seeded in 96-well plates at 2000 cells/well and incubated overnight. After the standard cell culture medium was replaced by the medium mentioned in the HSC migration assay, HSCs were incubated for another 24 h. After removing the culture medium, CCK-8 solution, diluted with FBS-free high-glucose DMEM at a ratio of 1:9, was added to each culture well and incubated for 1 h. Optical density (OD) was determined at 450 nm using a microplate reader (Bio-Rad Model 550, CA, USA). Relative cell proliferation rate was calculated as follows: [(OD CM treated group – OD blank group)/(OD control group – OD blank group)] × 100%. Each group was performed in sextuplicate and repeated three times.

### Gel contraction assay

Contractility of HSCs was evaluated using collagen gel lattices in a plastic 24-well culture plate. Collagen gels were made by mixing type I rat-tail collagen (BD Bioscience, Bedford, MA), 10 × DMEM (Solarbio Science & Technology, Beijing, China) and 0.1 mol/L NaOH. The collagen solution was mixed with a HSC suspension to a final solution as collagen concentration of 1 mg/ml and cell density of 2 × 10^5^ cells/ml. Five hundred microliters of mixed collagen gel solution was added into each well of a 24-well culture plate and was incubated for 1 h at 37 °C to allow gelation. Collagen gel was exposed to a complete culture medium (0.5 ml/well) and incubated in an incubator overnight. Then, cells in collagen gel were cultured with a serum-free medium for 4 h. After washing 3 times with PBS, the collagen gel was detached from the periphery of the wall of each well using a 10-μl micropipette tip. The cell culture medium was replaced by serum as mentioned above, and then cells in collagen gel were incubated for another 24 h. Each gel was photographed and analyzed with Image-Pro Plus 5.0. Contractility of HSCs was measured as follows: 100%-gel surface area/well basal area × 100%.

### Phospho-protein profiling by phospho-antibody array

Lysates from LX-2 cells treated with CM-C, CM-C plus HBeAg (2 μg/ml), and CM-E were tested by the Phospho Explorer Antibody Array (PEX100, Full Moon Biosystems Inc.) according to the manufacturer’s protocol. The phosphorylation ratio of each marker in different groups was analyzed as previously described [23].

### Cytokine determination by magnetic multiplex assay

Inflammation cytokines in the cell-culture supernatants were assessed by a Magnetic Luminex Performance Assay (R&D Systems) following the manufacturer’s guidelines. A Luminex X-200 (Luminex, Austin, TX) was used to read the multiplex assay. Absolute cytokine concentrations were determined using 5-parameter logistic curve fits in the R&D Analyte Software.

### Prediction of surface accessible peptide

Emini surface accessibility prediction tool of the Immune Epitope Database (IEDB) was applied to predict the surface accessible peptide of the core domain of HBeAg (ΔRP-E) using default threshold level 1.0 [24].

### Statistical analysis

Statistical analysis was performed with SPSS v22.0 software (SPSS Inc, Chicago, USA). Continuous variable results were expressed as mean ± standard deviation and categorical variables as the frequency or percentage. Student’s *t* test or one-way analysis of variance (ANOVA) was used to analyze significant differences between groups according to at least three independent experiments. Intergroup differences for clinical data were assessed using the chi-squared test, Mann-Whitney test, and Student’s *t*-test appropriately. Relationships between the parameters were characterized using the Spearman correlation coefficients. *P* < 0.05 was considered to be statistically significant.

## Results

### TLR-2 is the direct binding receptor of HBeAg for macrophage activation

**Fig. 1:**
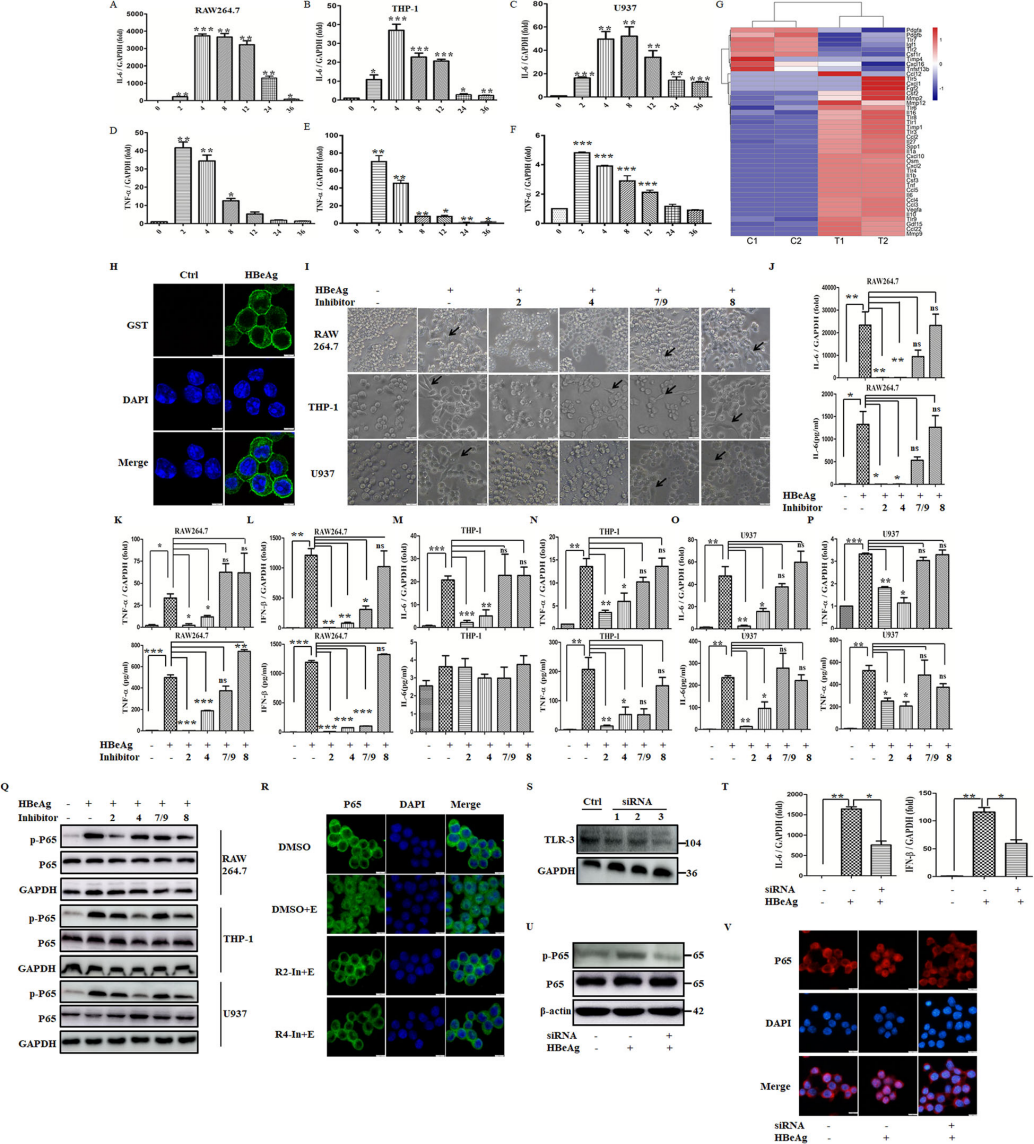
HBeAg regulated the expression of multiple TLRs in macrophages, while the inflammatory response of HBeAg-induced macrophages was inhibited by TLRs. RAW 264.7 macrophages were stimulated with HBeAg (2 μg/ml) at different time points, and then the expression of IL-6 and TNF-α was detected respectively using qRT-PCR (**A**, **D**). THP-1 (**B**, **E**) and U937 cells (**C**, **F**) were pretreated with PMA at a final concentration of 50 ng/ml. After replacing the fresh medium, they were stimulated with HBeAg (2 μg/ml) at different time points, and the expression of IL-6 and TNF-α was detected respectively using qRT-PCR. RAW264.7 macrophages were stimulated with HBeAg for 24 h, and RNA sequencing assay was performed. The expression profile of growth factors, cytokines, chemokines, and TLRs were selected and displayed (**G**). **H** After incubation with HBeAg for 1 h at 4 °C, RAW 264.7 macrophages were fixed with 1% paraformaldehyde for 20 min at room temperature. After blocking, cells were incubated with Alexa Flour 488-conjugated primary antibody for 1 h at 37 °C followed by DAPI for nuclear staining, then captured using a confocal fluorescence microscopy. RAW 264.7 macrophages and PMA-pretreated THP-1/U937 cells were treated with DMSO or inhibitors of TLR-2, 4, 8, 7/9 (10μΜ) for 30 min. The cell morphology was analyzed after HBeAg treatment for 24 h (**I**). The arrows indicate activated macrophages. RAW 264.7 macrophages were firstly treated with DMSO or inhibitors of TLR-2, 4, 8, 7/9 for 30 min, and then they were treated with HBeAg for 4 h. The expression and secretion of IL-6, TNF-α, and IFN-β were tested by qRT-PCR and ELISA respectively (**J**–**L**). PMA-pretreated THP-1 (**M**–**N**) and U937 cells (**O**–**P**) were treated with DMSO or inhibitors of TLR-2, 4, 8, 7/9 for 30 min, and then they were treated with HBeAg for 4 h. The expression and secretion of IL-6 and TNF-α were tested by qRT-PCR and ELISA respectively. Macrophages, including RAW264.7, PMA-pretreated THP-1 and U937 cells, were firstly treated with DMSO or inhibitors of TLR-2, 4, 8, 7/9 (10μΜ) for 30 min, then they were treated with HBeAg for another 20 min. The phosphorylation level of p65 was tested by western blot assay (**Q**). Additionally, the effect of TLR-2/4 inhibitors on the nuclear translocation of p65 was determined (**R**). The siRNAs for TLR-3 or negative control were transfected into RAW264.7 macrophages for 48 h, and then the cells were treated with HBeAg for 4 h. The expression of TLR-3 was tested by western blot assay (**S**), and the level of IL-6 and IFN-β (**T**) was tested by qRT-PCR. The siRNAs for TLR-3 or negative control were transfected into RAW264.7 macrophages for 48 h, and then the cells were treated with HBeAg for 20 min. The phosphorylation level and nuclear translocation of p65 were tested separately (**U**-**V**). *P < 0.05, **P < 0.01, ***P < 0.001

Our previous research has found HBeAg can induce macrophage activation [10]. To further analyze the recognition receptor of HBeAg in macrophages, we firstly detected the kinetics of typical cytokines at the dose of 2 μg/ml so as to determine the best detection time point (Fig. 1A–F). The expression of TNF-α and IL-6 in macrophages was induced at the earliest time point (2 h), and then maximal levels were reached approximately at 4 h after the stimulation of HBeAg. Therefore, the following data were detected at 4 h after HBeAg treatment. To understand the regulatory role of HBeAg on macrophage comprehensively, RNAseq analysis was applied. Notably, except for inflammation cytokines, a significant change of TLRs was also observed as demonstrated in the heat map (Fig. 1G). Therefore, the absolute expression of TLRs was analyzed in mouse and human macrophages by qRT-PCR to further verify this finding (Table 3). We found that TLR expression can be roughly divided into 3 levels: comparable high (TLR-2), medium (TLR-1, TLR-4, TLR-6, TLR-7, and TLR-9), or very low (TLR-3, TLR-5, and TLR-8). TLR-2 expression was upregulated most obviously in THP-1 cells pretreated with PMA (4.05 folds), whereas TLR-3 expression was increased most pronouncedly in RAW 264.7 macrophages (55.53 folds). As co-receptors of TLR-2, the expression of TLR-1 and TLR-6 was enhanced slightly (1–3 folds) in all of three cell lines. By contrast, HBeAg induced drastically reduction in expression levels of TLR-5 and TLR-7 (29–64%). Since the expression of TLR-5 in all three macrophage cell lines was extremely low and decreased significantly after HBeAg treatment, it indicates that its role in HBeAg recognition is negligible.

**Table 3:** The expression of TLR1-9 in RAW264.7, U937, and THP-1 cells

	RAW264.7			U937			THP-1		
	HBeAg(−)	HBeAg(+)	Fold	HBeAg(−)	HBeAg(+)	Fold	HBeAg(−)	HBeAg(+)	Fold
TLR1	7365	21,117	2.87	1432	1389	0.97	7853	12,121	1.54
TLR2	88,481	35,686	0.40	15,134	24,730	1.63	26,488	107,322	4.05
TLR3	254	14,105	55.53	18	21	1.17	189	101	0.53
TLR4	9555	9421	0.99	8342	6655	0.80	9205	6,977	0.76
TLR5	5	2	0.4	107	38	0.36	27	22	0.81
TLR6	772	1133	1.47	1110	1817	1.64	6921	8105	1.17
TLR7	32,821	25,970	0.79	64	35	0.55	541	380	0.70
TLR8	19	37	1.95	42	83	1.98	203	609	3.00
TLR9	2288	5013	2.19	167	255	1.53	564	1016	1.80

Note: The expression of TLRs was measured via Q-PCR. Copy numbers of TLR1-9 transcripts were normalized against GAPDH (×10^6^ copies GAPDH)

To define whether HBeAg can interact with TLRs, although it was able to regulate their expression, we firstly identified the location of HBeAg when it activated macrophages by immunofluorescence assay. As shown in Fig. 1H, HBeAg predominantly located at the cell boundaries, demonstrating that TLRs may play some roles in this process. Next, macrophages were pretreated with inhibitors of TLR-2 (C29), TLR-4 (resatorvid), TLR-7/9 (hydroxychloroquine sulfate), and TLR-8 (CU-CPT9b) for 30 min and subsequently treated with HBeAg. We observed the cell morphology and expression of different cytokines in order to screen the potential binding receptors. As displayed in Fig. 1I, macrophages pretreated with hydroxychloroquine sulfate and CU-CPT9b continued to become stretched and multilateral, indicating the activation of macrophages [25], while macrophages pretreated with C29 and resatorvid were not activated and kept their round morphology. Meanwhile, in accordance with the morphology, the levels of inflammatory cytokines in C29 and resatorvid pretreatment groups exhibited a significant reduction as compared with the group treated with HBeAg alone (Fig. 1J–P). Our previous studies verified that HBeAg promoted cytokine production via NF-κB signal pathway [10]. Therefore, we further tested the phosphorylation levels and nuclear translocation of p65 in macrophages treated with above-mentioned multiple inhibitors. As demonstrated in Fig. 1Q–R, macrophages pretreated with hydroxychloroquine sulfate and CU-CPT9b showed no or only slight attenuation in phosphorylation levels of p65. However, the phosphorylation and nuclear translocation of p65 were greatly declined in C29- or resatorvid-pretreated cells. Moreover, to evaluate the involvement of TLR-3 (no corresponding inhibitor), RAW264.7 macrophages were transfected with TLR-3-specific siRNAs. We confirmed a most evident reduction (>50%) of TLR-3 using seq-3 on day 2 after transfection (Fig. 1S), so seq-3 was used in the following analysis. And the expression of IL-6 and IFN-β was blocked dramatically (~ 50%) following the transfection of TLR-3-specific siRNAs (Fig. 1T). Consistently, the phosphorylation and nuclear translocation of p65 were also blocked as shown in Fig. 1U–V. Altogether, the above results signified that TLR-2/3/4 played crucial roles in the macrophage activation induced by HBeAg.

**Fig. 2:**
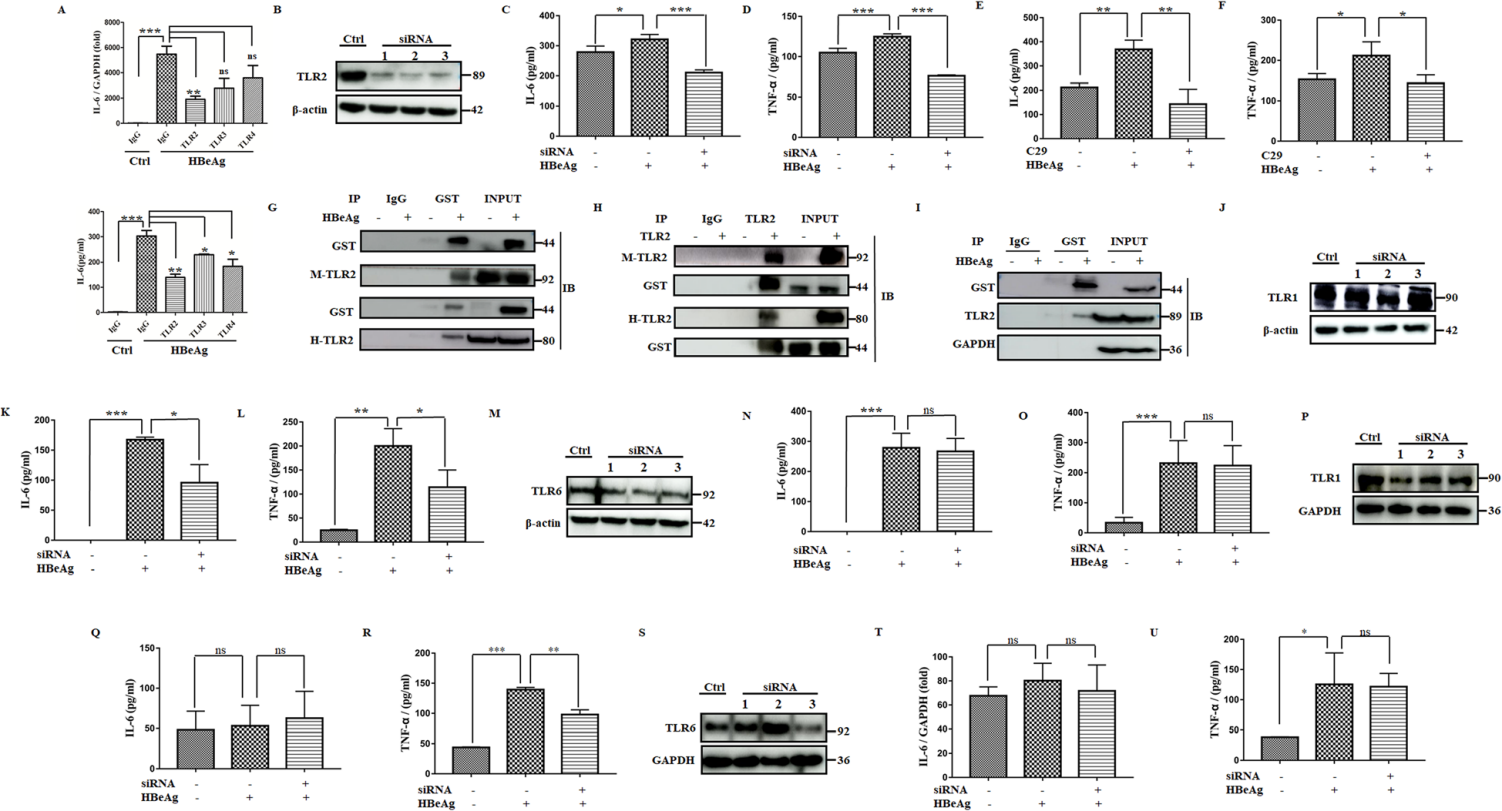
TLR-2 is the direct binding receptor of HBeAg when activating macrophages. After pretreated with isotype IgG or blocking antibodies for TLR-2/3/4 (5 μg/ml) for 30 min, RAW264.7 macrophages were stimulated with HBeAg for 4 h. The expression and secretion of IL-6 were analyzed by qRT-PCR and ELISA (**A**). The siRNAs for TLR-2 or negative control were transfected into hMDM for 48 h, and then the cells were treated with HBeAg for 4 h. The expression of TLR-2 was tested by western blot assay (**B**), and the level of IL-6 (**C**) and TNF-α (**D**) were tested by ELISA. Mouse Kupffer cells were treated with DMSO or inhibitors of TLR-2 for 30 min, and then they were treated with HBeAg for 4 h. The level of IL-6 (**E**) and TNF-α (**F**) were tested by ELISA. Co-IP analyzed the direct binding between recombinant GST-HBeAg and recombinant extracellular segment of TLR-2 proteins in vitro (**G**, **H**). RAW264.7 macrophages were treated with GST-HBeAg (2 μg/ml) for 1 h, and then the cells were lysed. Cell lysis was incubated with GST antibody or isotype IgG overnight at 4°C, and the direct binding between endogenous TLR-2 and recombinant GST-HBeAg was analyzed by western blot (**I**). The siRNAs for TLR-1, TLR-6, or negative control were transfected into RAW264.7 macrophages for 48 h, and then the cells were treated with HBeAg for 4 h. The expression of TLR-1 or TLR-6 were tested by western blot assay respectively (**J**, **M**), and the corresponding levels of IL-6 (**K**, **N**) and TNF-α (**L**, **O**) were tested by ELISA respectively. The siRNAs for TLR-1, TLR-6, or negative control were transfected into PMA-pretreated THP-1 macrophages for 48 h, and then the cells were treated with HBeAg for 4 h. The expression of TLR-1 or TLR-6 were tested by western blot assay respectively (**P**, **S**), and the corresponding levels of IL-6 (**Q**, **T**) and TNF-α (**R**, **U**) were tested by ELISA respectively. *P < 0.05, **P < 0.01, ***P < 0.001

Next, we used antibody blocking experiments for further verification. As displayed in Fig. 2A, the production of IL-6 was weakened most significantly by TLR-2-blocking antibody rather than TLR-3/4-blocking antibodies. Thus, we aimed at TLR-2 in the following analysis. Furthermore, we investigated the effect on hMDM using the siRNA knockdown of TLR-2. By day 2, there was a most significant decrease in TLR-2 protein expression using seq-2 (Fig. 2B). In accordance with the above results, the knockdown of TLR-2 prevented TNF-α and IL-6 production in the HBeAg stimulation group (Fig. 2C, D). Similar results could also be detected in mouse Kupffer cells pretreated with C29 (Fig. 2E, F). To determine whether HBeAg could interact with TLR-2 directly, we firstly performed co-immunoprecipitation assays with recombinant proteins of the extracellular segment of TLR-2. Our data showed that HBeAg could directly interact with TLR-2 in vitro (Fig. 2G, H). Furthermore, HBeAg was also able to interact with endogenous TLR-2 in vivo (Fig. 2I).

Finally, to distinguish which co-receptor is involved in TLR-2-mediated recognition and activation by HBeAg, we specifically knocked down TLR-1 and TLR-6 (Fig. 2J and M) and measured cytokines change. In RAW264.7 macrophages, the knockdown of targeted siRNAs for TLR-1 resulted in more significant inhibition of HBeAg-induced IL-6 and TNF-α production as compared with that of TLR-6 (Fig. 2K, L, N, O). Similar results for TNF-α induction were observed in PMA-pretreated THP-1 cell line (Fig. 2P–U). These results suggest TLR-1 may play a more important role in the activation of the HBeAg-induced TLR-2 signaling pathway.

### The C-terminal peptides of HBeAg played a key role in macrophage activation

**Fig. 3:**
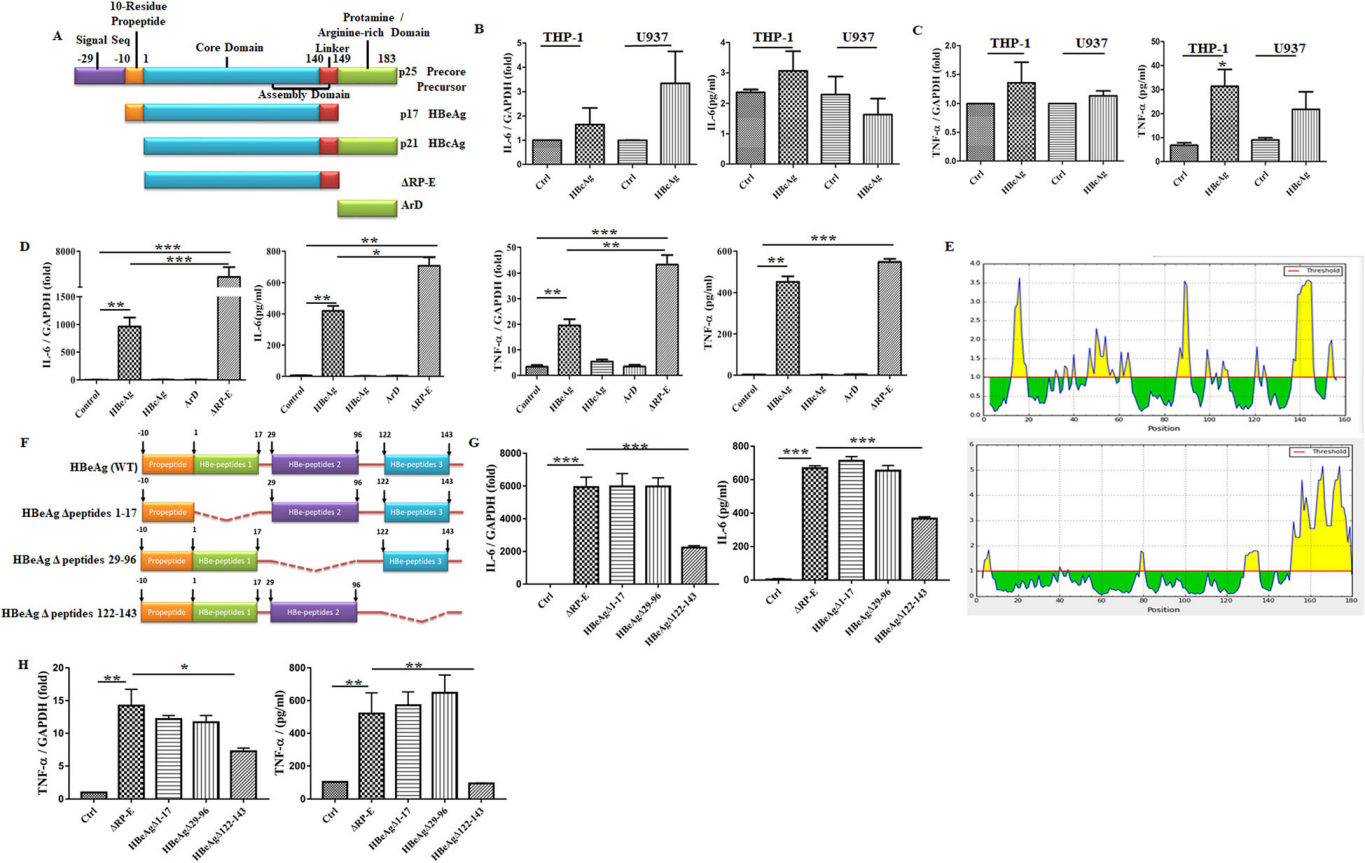
The C-terminal peptides of HBeAg played a key role in macrophage activation. Comparison of the structure for HBeAg, HBcAg, and their precore precursor proteins (**A**). PMA-pretreated THP-1/U937 cells were treated with 2 μg/ml HBcAg for 4 h, then the expression and secretion of IL-6 and TNF-α were tested by qRT-PCR and ELISA respectively (**B**, **C**). RAW264.7 macrophages were stimulated with HBeAg, HBcAg, ArD, and ΔRP-E (2 μg/ml) for 4 h respectively, and then the expression and secretion of IL-6 and TNF-α were tested by qRT-PCR and ELISA (**D**). Bioinformatics analysis of the surface accessible peptides for HBeAg (upper part) and HBcAg (lower part) was predicted (**E**). Schematic illustration of wild-type and truncation mutants of HBeAg (**F**). RAW264.7 macrophages were stimulated with ΔRP-E and truncation mutants of HBeAg (2 μg/ml) based on the analysis of the surface accessible peptides, then the expression and secretion of IL-6 and TNF-α were tested by qRT-PCR and ELISA (**G**, **H**). *P < 0.05, **P < 0.01, ***P < 0.001

HBeAg (p17) is processed from precore precursor protein (p25) with N- and C-terminal being truncated and shares a common core domain of 149 residues with HBcAg (p21) (Fig. 3A). However, the HBcAg is different from HBeAg based on our previous results, since it cannot significantly induce mouse macrophage activation [10]. To eliminate the effect that may be caused by species differences, we added recombinant HBcAg to PMA-pretreated THP-1 and U937 cells for 4 h. Consistently, we found the secretion and expression of IL-6 and TNF-α also exhibited no significant induction in both of these cell lines (Fig. 3B, C).

Next, we aimed to investigate the function domain of HBeAg for macrophage activation in order to further explain the immunogen difference between HBeAg and HBcAg. Accumulating data had shown that N-terminal propeptide of HBeAg determines the radically different molecular structure relative to HBcAg, and arginine-rich domain (ArD) of HBcAg is a prerequisite for the IL-18 production [26]. Thus, we synthesized recombinant protein ArD andΔRP-E (N-terminal 10-residue propeptide deleted HBeAg) and then treated macrophage with them. As shown in Fig. 3D, the expression and secretion of IL-6 and TNF-α were more increased in the group of ΔRP-E stimulation than that of full-length HBeAg, whereas ArD and HBcAg hardly triggered any changes compared with control. Moreover, we performed bioinformatics analysis for the surface accessible peptides, showing that the accessible peptides for HBeAg were scattered in three sections of the core domain (aa. 1-17, 29-96, 122-143), whereas for HBcAg it was concentrated in arginine-rich terminal (Fig. 3E). Thus, we speculated that the core domain of HBeAg was responsible for promoting the activation of macrophages, yet HBcAg adversely hided its immunogenicity due to extra ArD in this process. Furthermore, three recombinant HBeAg proteins which delete the above-mentioned accessible peptides were used to confirm their abilities in activating macrophages separately (Fig. 3F). Δaa.(122-143)-E was minimally stimulatory for macrophages compared with the others, suggesting that aa. 122-143 in HBeAg was crucial for macrophage activation (Fig. 3G, H).

### HBeAg promoted the activation of hepatic stellate cells in a macrophage-dependent manner

**Fig. 4:**
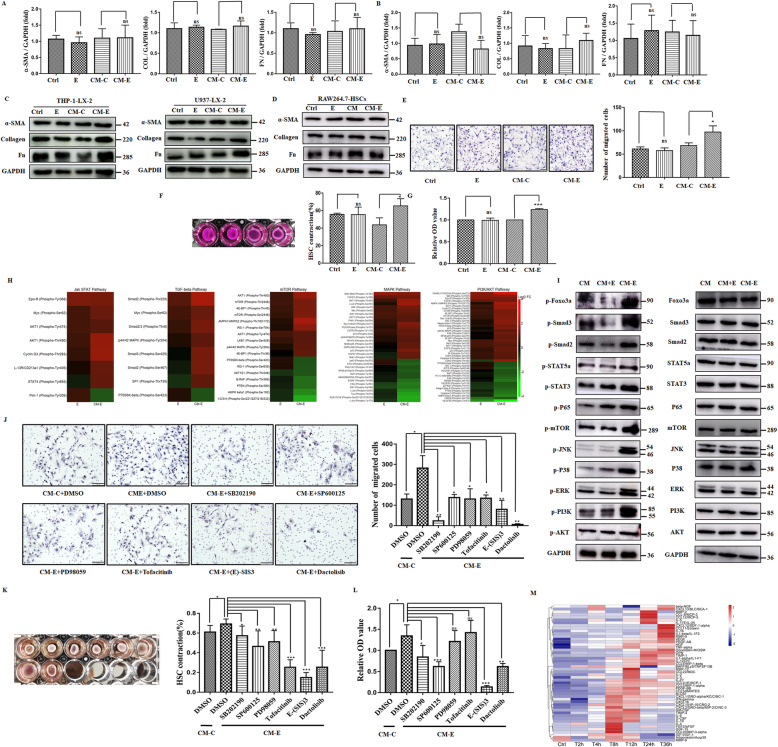
HBeAg promoted the activation of hepatic stellate cells in a macrophage-dependent manner. LX-2 stellate cells were treated with HBeAg (2 μg/ml), CM-C, or CM-E from RAW264.7 macrophages (**A**) or U937 cells (**B**) for 24 h, the expression of α-SMA, collagen 1a1, and fibronectin was tested by qRT-PCR. Similarly, their expression in LX-2 cells and primary hepatic stellate cells was tested by western blot assay (**C**, **D**). LX-2 stellate cells were treated with HBeAg (2 μg/ml), CM-C, or CM-E from U937 cells for 24 h; the contractility, proliferation, and migration phenotypes were detected (**E**–**G**). Log2-transformed phosphorylation signal of enriched pathways following treatment with HBeAg or CM-E was detected. The signal was normalized to respective total protein and then to CM control. Heat map: compared to vehicle, red indicates increased signal, black no change, and green reduced signal (**H**). Key signaling intermediates for enriched pathways of CM-E were verified using western blots (**I**). LX-2 cells were treated with CM and inhibitors of enriched pathways (10μΜ) or the same volume of DMSO as control, then the contractility, proliferation, and migration phenotypes were tested (**J**–**L**). RAW264.7 macrophages were stimulated with HBeAg (2 μg/ml) at different time points, and the secretion of cytokines, chemokines, and growth factors was detected using the Magnetic Luminex Performance Assay (**M**). *P < 0.05, **P < 0.01, ***P < 0.001

To analyze whether HBeAg can activate HSCs directly or in a macrophage-dependent manner, we added CM from HBeAg activated macrophages to human LX-2 stellate cells for 24 h compared with HBeAg alone. To our surprise, no significant difference of levels of α-SMA, collagen, and fibronectin was detected in different groups (Fig. 4A–C). The same results could also be detected from primary hepatic stellate cells treated with CM from RAW264.7 cells (Fig. 4D). In the ongoing process of hepatic fibrosis, activated HSCs exert pro-fibrosis effects not only by means of fibrillar extracellular matrix accumulation, but also by their proliferation, motility, and contractile phenotypes [27]. As shown in Fig. 4E, cells treated with CM-E exhibited the greatest chemotaxis toward serum, while cells treated with HBeAg did not display obvious chemotaxis compared with control. Additionally, the proliferation of HSCs also showed the same trend as the motility (Fig. 4G). Moreover, the surface area of the collagen lattice in control showed an evidently reduced, and the extent is similar with HSCs treated with HBeAg (Fig. 4F). In contrast, collagen lattice treated with CM-E caused more evident contraction as compared with lattice treated with CM-C.

### Conditioned medium from HBeAg-treated macrophages mediated HSC activation through different signal pathways

To investigate mechanisms that are regulated by CM-E for the activation of HSCs, we used a Phospho Explorer antibody microarray to detect and analyze phosphorylation events under specific conditions. Overall, we identified 92 proteins with at least 50% reduced, and 90 proteins with at least 100% increased phosphorylation. KEGG and pathway mapping analysis was further used to analyze these pivotal pathways. As compared with CM or CM with 2 μg/ml HBeAg, pathways that were significantly altered by CM-E were ErbB and mTOR signaling pathways, which mainly consist of MAPK, JAK-STAT, and PI3K-AKT signaling pathway. Besides, the TGF-β signaling pathway was the main pathway that was only affected by CM-E. The specific phosphorylation sites of the aforementioned pathways are illustrated in Fig. 4H, and key signaling intermediates were verified using western blots (Fig. 4I). To further reveal the signal pathway responsible for the HSC phenotype induced by CM-E, we pretreated LX-2 cells with the inhibitors of these pathways for 30 min and subsequently treated with CM-E. Though all of them may play some roles in the phenotype induced by CM-E, a more predominant effect was detected in PI3K-AKT-mTOR and p38 MAPK signaling pathway for motility (Fig. 4J) and the Smad-dependent TGF-β signaling pathway for proliferation and contraction of stellate cells (Fig. 4K, L). To analyze the soluble factors responsible for the phenotype, CM-E was screened for the level of various cytokines, chemokines, and growth factors using the Magnetic Luminex Performance Assay (Fig. 4M). Notably, soluble factors, such as CCL2, CCL5, CXCL10, and TNF-α, exhibited apparent secretion as early as 4 h after stimulation and were 9–137 folds higher than those produced by control macrophages. In addition, they have previously been reported to be key regulators of inflammation and liver fibrosis [15, 28]; hence, we have reason to believe that these soluble factors from macrophage supernatant induced by HBeAg may be key components of the activation of HSCs.

### HBeAg induced inflammation and fibrogenesis response in vivo via TLR-2 in macrophages

**Fig. 5:**
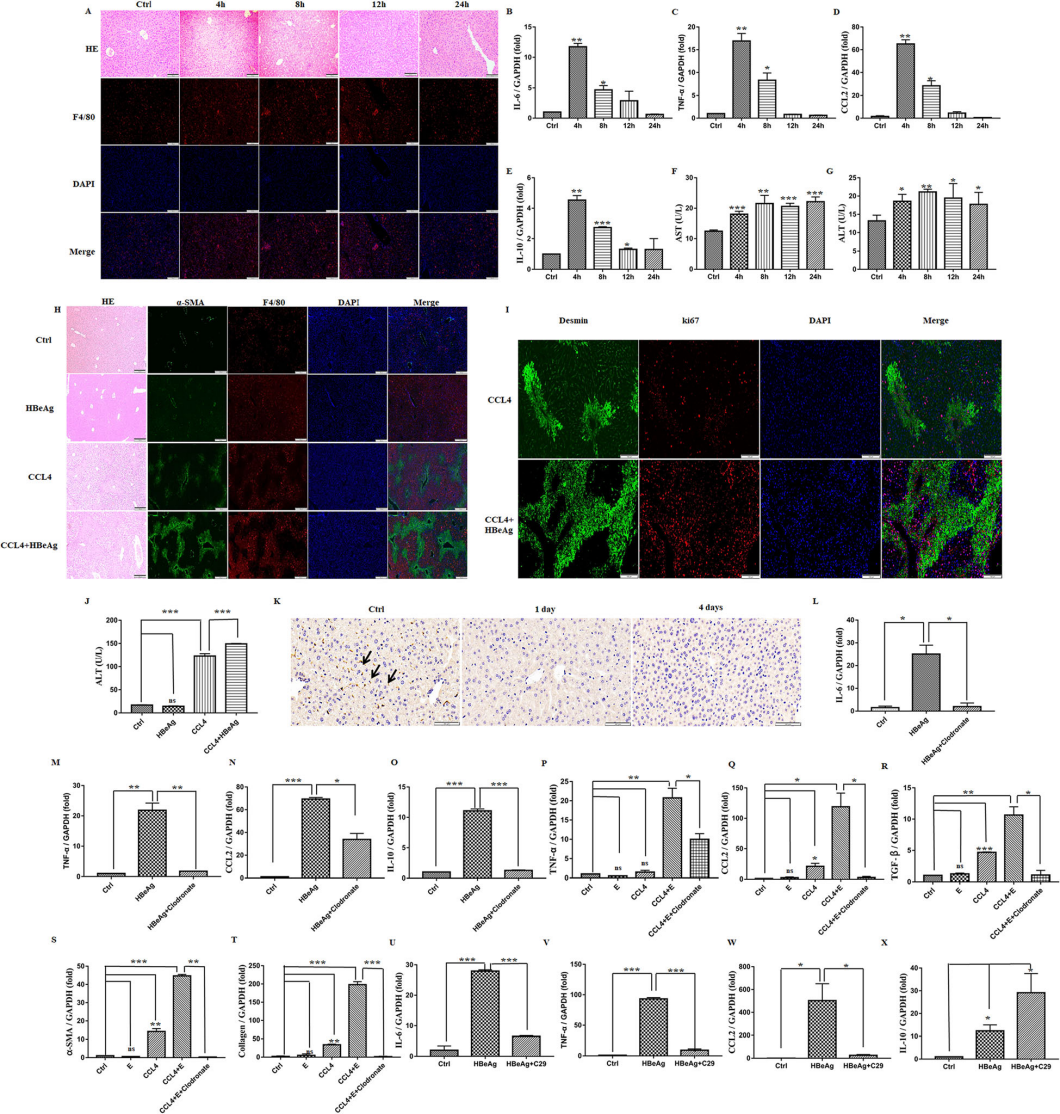
HBeAg induced inflammation and fibrogenesis response in vivo via TLR-2 in macrophages. Male Balb/c mice (5 mice in each group) were tail intravenous injected with recombinant HBeAg 40 μg or received the equivalent volume of PBS, then were sacrificed at 4, 8, 12, and 24 h respectively. Liver sections were stained with HE and F4/80 (**A**). The mRNA levels of the liver were detected via qRT-PCR (**B**–**E**). Plasma levels of ALT and AST were also examined (**F**, **G**). Male Balb/c mice (5 mice in each group) were treated with CCl_4_ (1 ml/kg in olive oil) together with intravenous administration of HBeAg 40 μg or the same volume of PBS. Liver sections were stained with α-SMA, F4/80, Desmin, and ki67 (**H**, **I**). Levels of serum ALT were examined (**J**). Male Balb/c mice were intraperitoneal injection of 0.2 ml clodronate-liposome (7 mg/ml) or control-liposome before CCl_4_ or HBeAg treatment as mentioned above. Monocyte depletion efficiency was examined at day 1 and day 4 by F4/80 staining (**K**). The mRNA levels in the liver of mice treated with HBeAg for 4 h were detected through qRT-PCR (**L**–**O**). The mRNA levels in the liver of mice treated with CCl_4_ and HBeAg in turn were tested by qRT-PCR (**P**–**T**). TLR-2 inhibitor (C29) was injected intraperitoneally (1.3 μmol/g) 1 h before HBeAg treatment (40 μg per mouse), with the same volume of dissolving reagent as the vehicle group (5 mice in each group). The mRNA levels in the liver were tested by qRT-PCR (**U**–**X**). *p = 0.05, **P < 0.01, ***P < 0.001

To analyze the immunoreaction of HBeAg in vivo, C57BL/6 mice were intravenous injected with recombinant HBeAg 40 μg for 4, 8, 12, and 24 h. As shown in Fig. 5A, there is a few small inflammatory infiltrates, but no observable hepatic necrosis areas (a common phenomenon of LPS and ConA-induced hepatitis) were detected at 4–12 h, which was in line with the transient expression of cytokines (Fig. 5B–E), implying that HBeAg is involved in the induction of hepatic inflammation. Concomitantly, both serum ALT and AST were increased mildly at different detection time (Fig. 5F, G). Additionally, the effect of HBeAg on fibrogenesis was assessed in the acute CCL_4_ model in mice. Co-immunofluorescence of F4/80 and α-SMA showed that HBeAg injection after CCL_4_ treatment aggravated macrophage infiltration and HSC activation. However, single HBeAg injection could not trigger HSC activation obviously (Fig. 5H). Meanwhile, HBeAg injection after CCL_4_ treatment contributed to the proliferation and motility of HSCs and elevated serum ALT (Fig. 5I, J). Therefore, HBeAg can promote the progression of liver injury and hepatic fibrosis rather than the leading cause of its initiation. To further elucidate the roles of macrophages in vivo, we depleted macrophages by injecting clodronate-containing liposomes before HBeAg or CCL_4_ treatment. Nearly all macrophages were depleted at day 1 and day 4 by clodronate as evidenced by reduced protein expression of F4/80 (Fig. 5K). Macrophage depletion led to a significant reduction in the expression of growth factors, cytokines, chemokines, α-SMA, and collagen in HBeAg treatment alone or the progression of fibrosis induced by CCL_4_ (Fig. 5L–T). Finally, to verify the roles of TLR-2 in vivo, mice were pretreated with C29 before the administration of HBeAg. We found the expression of IL-6, TNF-α, and CCL-2 was significantly alleviated (Fig. 5U–W), but IL-10 was upregulated in the liver (Fig. 5X).

### The level of HBeAg associated with inflammation and fibrosis degrees in patients infected with HBV

**Fig. 6:**
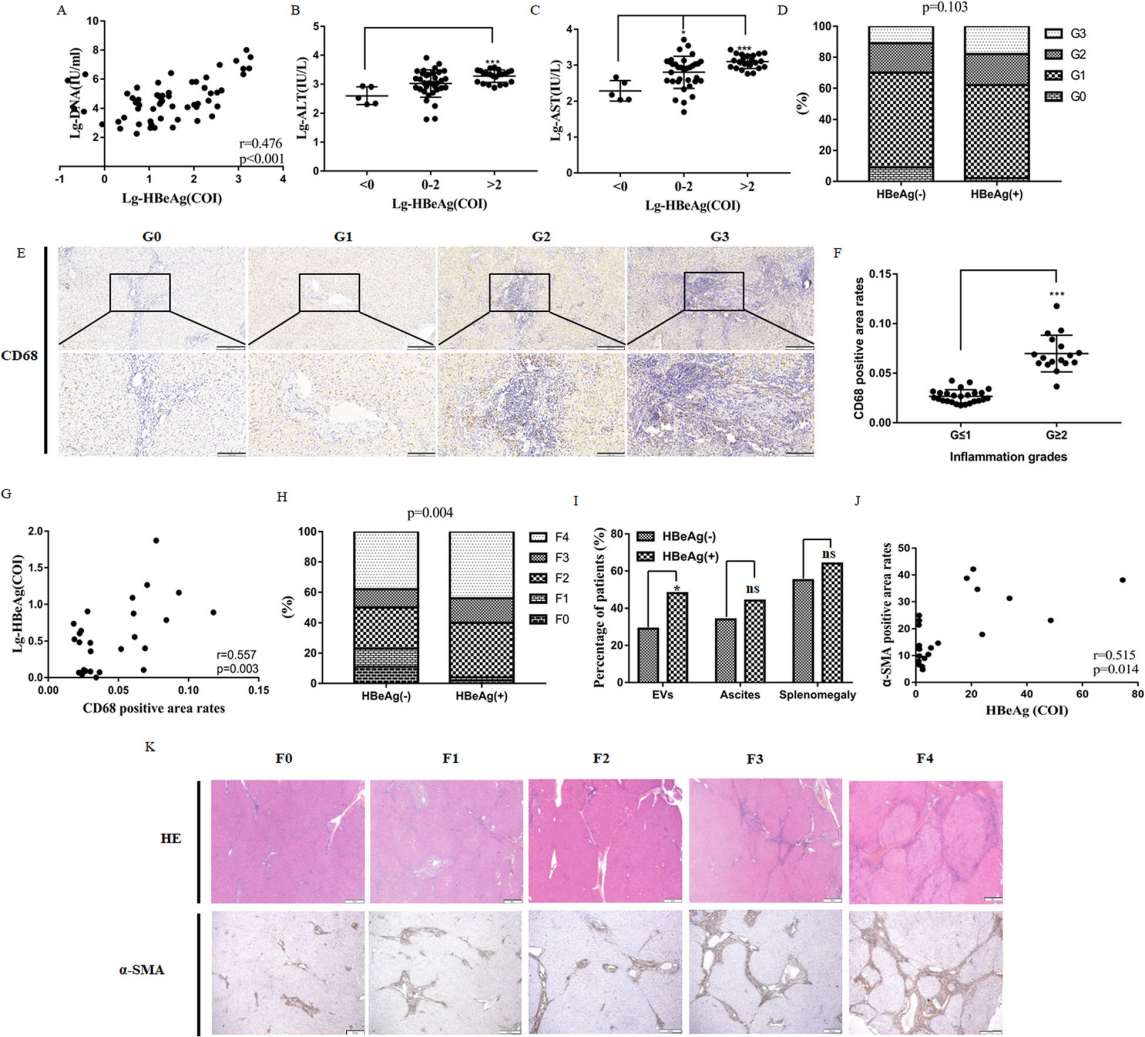
The level of HBeAg associated with inflammation and fibrosis degrees in patients infected with HBV. The association between the level of HBeAg and the content of HBV DNA (**A**), ALT (**B**), and AST (**C**) was analyzed in AHB patients. Hepatic inflammation grades were compared in HBeAg^−^ and HBeAg^+^ CHB patients (**D**). Liver sections were stained with CD68 (**E**), and CD68-positive areas were compared between CHB patients with significant inflammation grades (G ≥ 2) and their counterparts (G ≤ 1) (**F**). The association between the level of HBeAg and CD68-positive areas was analyzed (**G**). Hepatic fibrosis stages and the percentage of patients with CHB-related complications were compared in HBeAg^−^ and HBeAg^+^ patients (**H**, **I**). Liver sections were stained with HE and α-SMA (**K**), and the level of HBeAg and the α-SMA-positive areas was analyzed in 20 HBeAg^+^ patients (**J**). *P < 0.05, **P < 0.01, ***P < 0.001

To verify our results in patients infected with HBV, we recruited 61 patients with AHB and 151 patients with CHB. As an index of viral replication, the level of HBeAg was positively correlated with HBV DNA load in AHB patients (Fig. 6A). Moreover, the levels of serum ALT, AST were increased with the HBeAg in these patients (Fig. 6B, C).

Next, we analyzed the relationship between the level of HBeAg and fibrosis stages and inflammation grades in CHB patients. As shown in Fig. 6D, hepatic inflammation grades were higher in HBeAg^+^ patients than HBeAg^−^ patients; nevertheless, statistical significance could only be achieved between G = 0 and G ≥ 1 (*P* = 0.030). CD68, a marker for monocytes and macrophages, was estimated by immunohistochemical analysis in all of HBeAg^+^ patients (*n* = 45) (Fig. 6E). The positive area of CD68 was higher in patients with significant inflammation grades (G ≥ 2) (Fig. 6F), indicating its close relationship with hepatic inflammation activity in CHB patients. Meanwhile, a significant correlation was observed between the CD68^+^ cell infiltration and the level of HBeAg (*r* = 0.557, *P* = 0.003, Fig. 6G). Moreover, hepatic fibrosis was more severe in HBeAg^+^ patients than HBeAg^−^ patients (*P* = 0.004, Fig. 6H). Consistently, HBeAg^+^ patients showed a higher incidence of the cirrhosis-related complications such as esophageal varices (EVs), ascites, and splenomegaly, even though only rates of EVs achieved statistical significance (Fig. 6I). Furthermore, α-SMA was detected for 20 HBeAg^+^ patients (Fig. 6K). As demonstrated in Fig. 6J, its positive staining areas were correlated with the level of HBeAg positively (*r* = 0.515, *P* = 0.014). Taken together, we demonstrated that HBeAg may act as a vital regulator of hepatic inflammatory and fibrosis response in patients infected with HBV.

## Discussion

HBV infection is an important public health issue. The major harm of HBV infection lies in the persistent liver injury caused by the activation of the immune system, and concomitant wound-healing response, which will finally lead to the occurrence of hepatic cirrhosis and its related complications. Macrophages, as an important component of the innate immune system, represent the first line of defense against pathogens in the liver. During infection, macrophages can sense the presence of HBV pathogen-associated molecular patterns through PRRs, and further secrete a large spectrum of inflammatory cytokines to mediate inflammation response. Moreover, macrophages are also able to prompt an effective adaptive immunity and contribute to a vigorous T cell response as well as B cell-mediated antibody secretion. In addition, liver fibrosis is induced by sustained low-grade injury during chronic HBV infections, and one of the most important mechanisms for its formation is the crosstalk between HSCs and macrophages in a paracrine manner [29]. Accumulating data have showed that macrophages play vital roles in both the injury and recovery phases of inflammatory scarring [30].

Sensing and responding to pathogens for macrophages are largely mediated by PRRs, including scavenger receptors, TLRs, RIG-like receptors, NOD-like receptors, and C-type lectins [31]. HBV particles and its related proteins could be detected and recognized by macrophages, thus activating surface and/or intracellular receptors to produce viral inhibitory cytokines [8]. Recently, only limited information exists in the direct interaction between HBV with macrophages in vivo and in vitro. It has been reported that TLR-2 and heparan sulfate proteoglycan were responsible for HBcAg recognition, thereupon resulting in the production of IL-6, IL-12p40, and TNF [5]. However, HBcAg only existed within infected hepatocytes or viral particles, and our data have confirmed that HBcAg cannot induce macrophage activation significantly in mouse or human macrophages. Thus, there might be other ways for macrophages to recognize HBV. Moreover, HBsAg can stimulate human blood monocytes in a CD14-dependent manner [32], and the complex formation with albumin may increase its uptake by Kupffer cells [33]. Intriguingly, for dendritic cells, HBsAg internalization is mediated through the mannose receptor [34], so we may speculate the same viral protein is able to trigger immune response at diverse binding sites. The available evidence shows the interaction of HBeAg with mIL-1RAcP may trigger host IL-1 response by activating downstream NF-κB signal pathway by means of IκB degradation [35]. In this study, we further verified that TLR-2 is the direct binding receptor of HBeAg when activating macrophages both in vivo and in vitro, and C-terminal peptides of HBeAg serve as molecular basis for the activation of macrophages induced by HBeAg. This finding provides a novel mechanism in HBV-induced macrophage activation.

On the other hand, HBV particles and its related proteins may repress immune response to a certain extent and contribute to persistent infection. Intriguingly, there are species-specific differences in this respect, which are mainly characterized by the difference of the expression of TLRs and their regulation induced by HBeAg in our study. In an un-activated state, the expression of several TLRs in mouse macrophage was higher than that in human macrophage, especially for TLR-2, TLR-7, and TLR-9. Therefore, our results indicated a more pronounced inflammation response in mouse macrophages compared with human macrophages, and this finding may explain that HBV can replicate persistently in human livers but not in mice under natural conditions. Moreover, some excellent reviews also specified that the differences in the structure, sequences, and localization patterns of TLRs may also determine divergent functions across different species [36–38]. Additionally, the mechanism of immune escaping induced by HBeAg might not be the same between mice and humans. We observed that TLR-2 displayed the highest expression in mouse macrophages, while its expression reduced most significantly after HBeAg stimulation. However, for human macrophages, HBeAg may mainly decrease the expression of TLR-3 which is associated with the IFN production. Overall, our results also verified the previous finding that HBV can downregulate TLR expression so as to inhibit the antiviral response from macrophages and facilitate immune tolerance [29]. Moreover, HBV replication could be abolished through the treatment of ligands for TLR-7 and TLR-5, implying these receptors may play pivotal roles in inhibiting HBV replication [39]. However, both of them were downregulated in both mouse and human macrophages treated with HBeAg, suggesting another novel mechanism of immune escaping and tolerance induced by HBV. Aiming at the same goal, HBV may also facilitate anti-inflammatory responses in vivo directly. Kupffer cells in HBV-carrier mice expressed more IL-10 and mediated the systemic tolerance induction in an IL-10-dependent manner [40]. Consistently, at the peak of viremia, during the inhibition of lymphocyte activation, a peak of IL-10 was observed in the serum of patients infected with acute HBV [41]. In this study, HBeAg also induces the expression of IL-10 in the liver, while IL-10 was upregulated more pronouncedly in mice pretreated with TLR-2 inhibitors, indicating HBeAg may also target other signal pathway mediators in this process. Thus, the expression of TLR-2 may resist the formation of immune tolerance during the HBV infection. Consistently, primary human hepatocytes have recently been reported to sense HBV particles through TLR-2, leading to an activation of anti-HBV immune responses in vitro [42]. In the future, more attention should be paid in the mechanism and clinical significance in this field. Finally, Lang et al. find propeptide of HBeAg is identified as similar with the TIR motif, thereby suppressing TIR-mediated activation of the inflammatory responses by disrupting homotypic TIR:TIR interaction [43]. Accordingly, HBeAg is the most crucial protein in HBV-associated antigen activating macrophages, and the core domain (ΔRP-E) triggered more expression and secretion of cytokines when comparing with HBeAg and HBcAg. In addition, C-terminal ArD is unique and the only surface accessible domain for HBcAg, and the previous study indicates HBcAg may promote cytokine induction from macrophages in a ArD-dependent manner [5]. However, neither HBcAg nor ArD can induce macrophage activation significantly in this study, and we speculate ArD, just like propeptide of HBeAg, may also play a crucial role in pro-inflammatory secretion inhibition. Altogether, it seems that activation and inhibition of immune response may co-exist in the process of HBV infection.

In HBV-infection microenvironments, activated macrophages are proved to be involved in liver fibrosis by activating HSCs directly or indirectly [31]. Actually, increased numbers of CD16^+^ and CD163^+^ macrophages correlated with higher histological activity and fibrosis degrees in CHB patients [22, 44], whereas the causative role of macrophages in the development of HBV-related fibrosis has not been elucidated. To determine the role of HBeAg in the pathogenesis of HBV-related fibrosis, we used serum-free CM to stimulate HSCs, since serum contains growth factors and cytokines which may mask potential effects produced by macrophage-derived mediators on hepatic stellate cells [45]. We found a macrophage-dependent way induced by HBeAg to enhance the proliferation, contraction, and motility of HSCs. Consistent with findings in vivo, HBeAg represent a mediator of perpetual fibrogenesis, which is characterized by promoting the activation of HSCs, thereby exacerbating hepatic fibrosis. On the contrary, HBeAg alone cannot activate HSCs directly, even though HSCs do express TLR-2. This phenomenon could be ascribed to the extremely low expression of TLR-2 in HSCs (Table 3 and Additional file 1: Table S1). Moreover, the expression of TLR-2 in HSCs remained almost unchanged after being treated with HBeAg. Overall, HSCs and macrophages may play different roles in recognizing HBeAg, and the activation of HSCs induced by HBeAg requires the help of macrophages. Furthermore, we explored signal pathways responsible for these phenotypes. The Smad-dependent TGF-β signaling pathway is generally considered to be the most effective fibrogenic pathway [15], in which signal transducing into the nucleus is mediated by phosphorylated receptor-activated Smads. Besides, many studies also identified TGF-β exerts its actions via crosstalk with other signal pathways in a Smad-independent way, such as mitogen-activated protein kinase (MAPK), mammalian target of rapamycin (mTOR), phosphatidylinositol-3-kinase/AKT, and Rho GTPase pathways [46]. In this study, phenotypes of activated HSCs induced by HBeAg were often regulated by multiple pathways. The Smad-dependent TGF-β signaling pathway is responsible for the proliferation and contraction of stellate cells, while PI3K-AKT-mTOR and p38 MAPK pathways may play a more vital role in the motility of HSCs. These data demonstrate that the activation of HSCs may be regulated through both Smad-dependent and Smad-independent pathways, and further study should verify this conclusion with Smad2 and Smad3 knockout models. Except for TGF-β, various growth factors, cytokines, and chemokines, such as IL-6, TNF-α, IL-1, PDGF, and CCL2, may also serve as messengers released from macrophages to regulate phenotypes of HSCs. Therefore, this specific crosstalk between macrophages and HSCs via these soluble proteins can be an aspect of further research.

Clinically, the progression of hepatic fibrosis caused by continuous inflammation response is an important factor of poor prognosis in CHB patients. HBeAg status has usually been identified as a significant serum marker to differentiate the natural history stage of CHB. Currently, it remains controversial with respect to the association between the HBeAg level and pathological fibrosis/inflammation degrees. In this study, we demonstrated that the HBeAg^+^ status was related to higher inflammation or fibrotic degrees in CHB patients which was in accordance with previous studies [47–49]. Notably, the average age of recruited CHB patients is 54 years old, so we can exclude the vast majority of patients with immune tolerance who are always less than 30 years and characterized with HBeAg positivity but no significant immune response to the virus [50]. Moreover, a more close relationship was observed between HBeAg status and mild hepatic inflammation grades (G ≥ 1); thus, we surmised HBeAg may play a more important role in inducing the early inflammation response. However, other HBV-related components as mentioned above may lead to the persistent inflammatory response. Even though a few studies have established HBeAg^+^ status was independently associated with hepatic significant fibrosis [47], we further revealed that the concentration of serum HBeAg was increased with CD68 and α-SMA-positive areas in HBeAg^+^ patients. Therefore, the clinical data verified our findings that HBeAg promoted hepatic fibrosis via a macrophage-dependent manner.

**Fig. 7:**
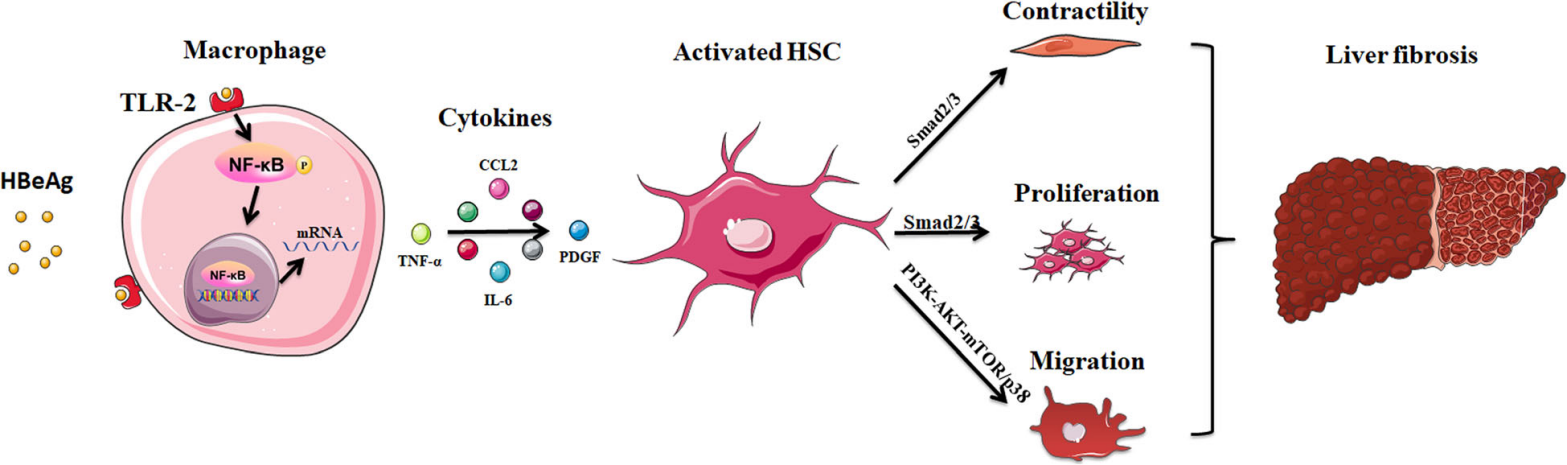
Schematic diagram of HBeAg-mediated inflammatory functions of macrophages via TLR-2 contributing to hepatic fibrosis through stellate cell activation

In summary, our results have indicated that HBeAg mediated the innate immune response induced by macrophages through affecting the expression of TLRs and signal pathway activation. Moreover, we validated that TLR-2 was the direct binding receptor of HBeAg, and C-terminal peptide (122-143 aa.) of core domain in HBeAg was critical for macrophage activation. Furthermore, HBeAg promoted the proliferation, contraction, and motility of HSCs in a macrophage-dependent manner. Mechanically, PI3K-AKT-mTOR and p38 MAPK signal pathway contributed to the motility of HSCs, while the Smad-dependent TGF-β signal pathway promoted their proliferation and contraction. Additionally, soluble factors, such as CCL2, CCL5, CXCL10, and TNF-α may be responsible for these above phenotypes. In vivo, we further verified that HBeAg may play a more important role in the early inflammation response and promote the progression of hepatic fibrosis. Taken together, we unveiled a novel interaction between HBV infection and innate immune response via TLRs and further expanded the understanding of HBV-induced hepatic fibrogenesis mechanism (Fig. 7).

## Conclusions

HBeAg activated macrophages via the TLR-2/NF-κB signal pathway, and further exacerbated hepatic fibrosis by facilitating motility, proliferation, and contraction of HSCs with the help of macrophages.

## Supplementary Information

**Additional file 1.** The expression of TLR-2 in LX-2 cells.

## Data Availability

The data that support the findings of this study are available from the corresponding author upon reasonable request.

## Abbreviations

AHB: Acute hepatitis B
ANOVA: One-way analysis of variance
ArD: Arginine-rich domain
CCK-8: Cell Counting Kit-8
CHB: Chronic hepatitis B
CM: Conditioned medium
CM-C: Conditioned medium collected from control
CM-E: Conditioned medium collected from activated macrophages by HBeAg
Co-IP: Co-immunoprecipitation
ECM: Extracellular matrix
ELISA: Enzyme-linked immunosorbent assay
EVs: Esophageal varices
HBcAg: Hepatitis B core antigen
HBcrAg: Hepatitis B core-related antigen
HBeAg: Hepatitis B e antigen
HBsAg: Hepatitis B surface antigen
HBV: Hepatitis B viruses
HCC: Hepatocellular carcinoma
HSC: Hepatic stellate cell
IEDB: Immune epitope database
OD: Optical density
PRR: Pattern recognition receptor
qRT-PCR: Quantitative real-time PCR
TLR: Toll-like receptor
