# Correction: HBeAg mediates inflammatory functions of macrophages by TLR2 contributing to hepatic fibrosis

**DOI:** 10.1186/s12916-022-02392-3

**Published:** 2022-05-04

**Authors:** Xiaoyu Xie, Huanran Lv, Chenxi Liu, Xiaonan Su, Zhen Yu, Shouyang Song, Hongjun Bian, Miaomiao Tian, Chengyong Qin, Jianni Qi, Qiang Zhu

**Affiliations:** 1 Shandong Provincial Hospital Affiliated to Shandong First Medical University, Jinan, Shandong 250021 People’s Republic of China; 2 Shandong Provincial Hospital, Cheeloo College of Medicine, Shandong University, Jinan, Shandong 250021 People’s Republic of China; 3 Shandong Provincial Engineering and Technological Research Center for Liver Diseases Prevention and Control, Jinan, Shandong 250021 People’s Republic of China; 4 The First Affiliated Hospital of Xinjiang Medical University, Urumqi, Xinjiang 830054 People’s Republic of China

**Correction: BMC Med 19, 1-20 (2021)**

**https://doi.org/10.1186/s12916-021-02085-3**

After publication, it came to the authors’ attention that after review of the figures in our manuscript [1], Figure 1Q and Figure 2T were displayed incorrectly.

- The p-P65 western blot strip of U937 macrophages (original Figure 1Q) were obtained from an uncorresponding file. Figure 1Q has been amended and the correct version can be viewed as the corrected Figure 1Q. 
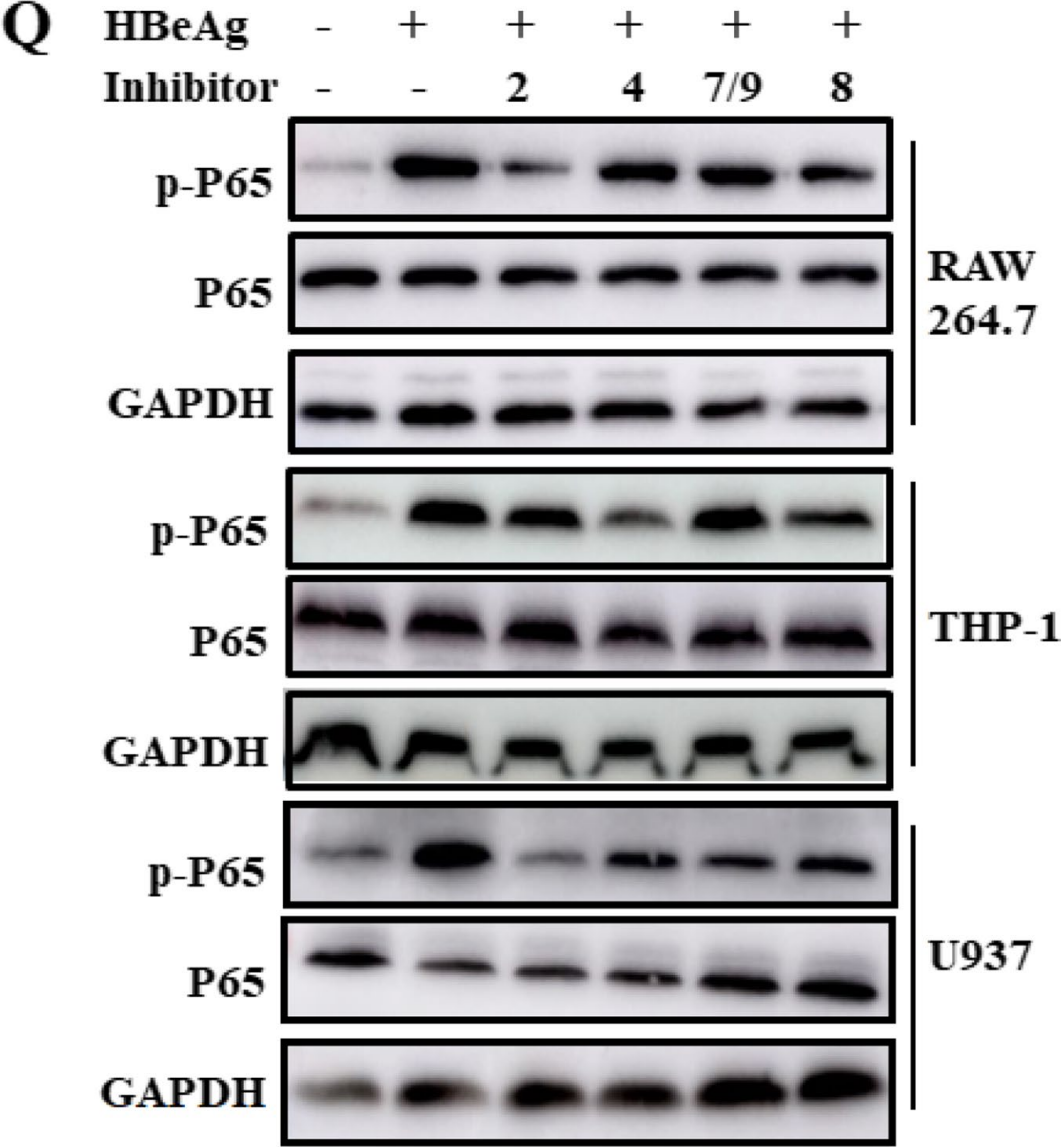


- Additionally, in the original manuscript, Figure 2T was incorrectly labelled on the Y-axis as “IL-6 / GAPDH (fold)”. It should be correctly labelled as “IL-6 (pg/ml) ”. Figure 2T has been amended and the correct version can be viewed as the corrected Figure 2T. 
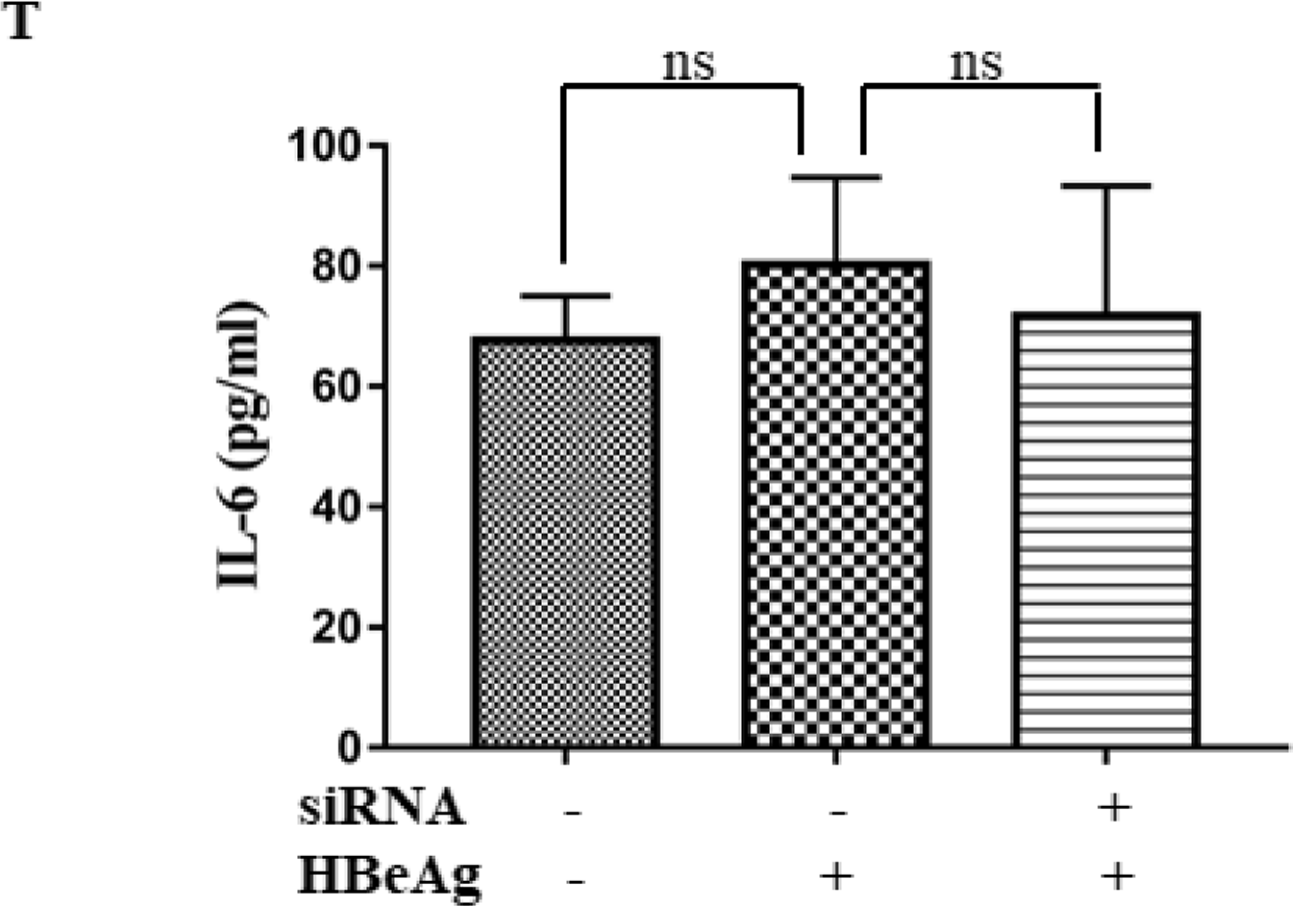


The figures corrected in this erratum do not influence any original conclusions in this study. We apologize for any inconvenience or misunderstanding that the errors may have caused.
