# Supplementary material for: HBeAg mediates inflammatory functions of macrophages by TLR2 contributing to hepatic fibrosis
Source: BMC Med. 2021 Oct 15;19:247. doi: 10.1186/s12916-021-02085-3 (PMC8518250; doi:10.1186/s12916-021-02085-3)
Supplement: Supplementary file 1 — Additional file 1. The expression of TLR-2 in LX-2 cells. [file 12916_2021_2085_MOESM1_ESM.docx]

***Table-S1.*** The expression of TLR-2 in LX-2 cells.

| LX-2 | | | |
| --- | --- | --- | --- |
|  | HBeAg(-) | HBeAg(+) | fold |
| TLR-2 | 44 | 45 | 1.02 |

Note: The expression of TLR-2 was measured via Q-PCR. Copy numbers of TLR-2 transcripts were normalized against GAPDH (×10^6^ copies GAPDH).
